# Association of Ang/Tie2 pathway mediators with endothelial barrier integrity and disease severity in COVID-19

**DOI:** 10.3389/fphys.2023.1113968

**Published:** 2023-02-21

**Authors:** Carla Roberta Peachazepi Moraes, Ivanio Teixeira Borba-Junior, Franciele De Lima, Jéssica Ribeiro Alves Silva, Bruna Bombassaro, André C. Palma, Eli Mansour, Lício Augusto Velloso, Fernanda Andrade Orsi, Fábio Trindade Maranhão Costa, Erich Vinicius De Paula

**Affiliations:** ^1^ School of Medical Sciences, University of Campinas, Campinas, Brazil; ^2^ Obesity and Comorbidities Research Center, University of Campinas, Campinas, Brazil; ^3^ Institute of Biology, University of Campinas, Campinas, Brazil; ^4^ Hematology and Hemotherapy Center, University of Campinas, Campinas, Brazil

**Keywords:** endothelial barrier, COVID-19, angiogenesis, angiopoietin, VEGFA

## Abstract

Endothelial barrier (EB) disruption contributes to acute lung injury in COVID-19, and levels of both VEGF-A and Ang-2, which are mediators of EB integrity, have been associated with COVID-19 severity. Here we explored the participation of additional mediators of barrier integrity in this process, as well as the potential of serum from COVID-19 patients to induce EB disruption in cell monolayers. In a cohort from a clinical trial consisting of thirty patients with COVID-19 that required hospital admission due to hypoxia we demonstrate that i) levels of soluble Tie2 were increase, and of soluble VE-cadherin were decreased when compared to healthy individuals; ii) sera from these patients induce barrier disruption in monolayers of endothelial cells; and iii) that the magnitude of this effect is proportional to disease severity and to circulating levels of VEGF-A and Ang-2. Our study confirms and extends previous findings on the pathogenesis of acute lung injury in COVID-19, reinforcing the concept that EB is a relevant component of this disease. Our results pave the way for future studies that can refine our understanding of the pathogenesis of acute lung injury in viral respiratory disorders, and contribute to the identification of new biomarkers and therapeutic targets for these conditions.

## 1 Introduction

In COVID-19, acute respiratory distress is mediated by the activation of several components of the inflammatory response in a process referred to as immunothrombosis (Engelmann and Massberg, 2013; Bonaventura et al., 2021). This process involves platelet activation, generation of NETs (neutrophil extracellular traps), tissue factor expression, complement and endothelial activation, which are important elements for pathogen eradication and tissue repair, but that can also mediate tissue damage if activated in a deregulated fashion. Regulation of endothelial barrier (EB) integrity is an essential step of this process, as the disruption of the endothelial line is required for the access of leukocytes to tissues during diapedesis (Seeley et al., 2012).

The physiological mechanisms responsible for EB integrity have been studied for more than a century, with Ernest Starling publishing his model of microvascular fluid dynamics in 1896 which was largely based on differences of oncotic pressure between the intravascular and interstitial space (Starling, 1896; Jacob and Chappell, 2013). During the last century several new concepts were added to our understanding of this process such as the role of the glycocalyx (Michel, 1979) and the discovery of humoral mediators of EB integrity, first identified in studies of angiogenesis during embryonic life (Lomas et al., 1971; Ferrara et al., 2003; Byrne et al., 2005), but later shown to be involved in EB integrity regulation during inflammation. These mediators include VEGF-A and angiopoietins (Ang) 1 and 2, as well as their receptor Tie2 (Gavard and Gutkind, 2006; Schliemann et al., 2006; Park-windhol and Amore, 2016). Interestingly, levels of some of these mediators have been shown to be predictors of disease severity in sepsis (Pickkers et al., 2005; Marx et al., 2013; Page and Liles, 2013). Accordingly, angiogenesis and EB disruption are two intertwined process that can participate in the host response to an infection. Both angiogenesis and EB disruption have been shown to participate in the pathogenesis of COVID-19. The former is evidenced by the clinical observation of intra-alveolar fluid accumulation which is one of the hallmarks of lung pathology in this disease. The latter has been evidenced in human pathology samples showing that intusseptive angiogenesis is present in COVID-19, along with disruption of intercellular junctions (Ackermann et al., 2020), which is also accompanied by evidences of structural destruction of endothelial cell membranes (Magro et al., 2021). Finally, levels of some of the main mediators of EB disruption such as VEGF-A and Ang-2 have been associated with clinical severity in COVID-19 (Pine et al., 2020; Smadja et al., 2020; Rovas et al., 2021; Schmaier et al., 2021; Smadja et al., 2021).

Here we investigated whether levels of components of the Ang/Tie2 pathway and of VEGF-A are associated with EB disruption and with clinical severity in patients with COVID-19.

## 2 Materials and methods

### 2.1 Study design and study population

The study was performed using samples from a previously described population (Henderson et al., 2022), recruited as part of a clinical trial conducted at a tertiary academic hospital from University of Campinas (Mansour et al., 2021). Samples were obtained prior to any intervention, within 24 h from the confirmation of COVID-19 by RT-PCR. Patients were recruited consecutively, and inclusion criteria included the need for hospital admission due to hypoxia and evidence of lung disease in lung CT scans. A group of healthy individuals matched by sex and age was recruited concomitantly from the same geographic area. The study was performed in accordance with the Declaration of Helsinki and approved by the local IRB (protocol CAAE: 36528420.3.0000.5404).

### 2.2 Sample collection and processing

Whole blood samples were collected into EDTA K2 and serum tubes immediately after study enrollment. Samples were processed within 2 h from collection and centrifugation at 2,500 g for 15 min at 22°C for EDTA K2 for plasma, and 1,000 g for 10 min at 10°C for serum. Samples divided in 300 µL aliquots, and immediately frozen at −80°C until analysis.

### 2.3 Clinical and laboratory data

Clinical and laboratory data were obtained from electronic medical records and from case report forms of the clinical trial.

### 2.4 Levels of EB mediators

Plasma levels of EB mediators levels were performed by immunoenzymatic method (ELISA) using commercial kits (R&D) for sTie2 and soluble VE-cadherin (sVEC), and by a customized Luminex immunoassay (Procarta Plex multiplex panel, Thermo-Fischer Scientific) for Ang-1 and VEGF-A; or Ang-2 (Human Angiogenesis/growth factor magnetic bead panel 1 - Merck Millipore), in a Bioplex 200 instrument (Bio-Rad).

### 2.5 Cell culture

Human umbilical vein endothelial cells HUVECs were grown in 75 cm^2^ flasks in EBM-2 medium supplemented with 10% fetal bovine serum at 37°C in an atmosphere of 5% CO2/95% air. Medium was replaced every 48 h until sub-confluency (approximately 80%–90%) was reached. Human lung endothelial cells (HULECs) were grown in 75 cm^2^ flasks in DMEM medium supplemented with 10% fetal bovine serum. Cultivation conditions were the same used for HUVECs cells. Medium was replaced every 48 h until reaching the estimated confluence of 80%. All experiments performed on HUVECs and HULECs were between passages 5–7. HUVECs were obtained from Lonza (Walkersville, MD, United States) and HULECs were obtained from ATCC (Manassas, VA, United States).

### 2.6 *In Vitro* evaluation of endothelial barrier function

EB integrity was assessed using ECIS, an electrical cell substrate impedance detection system (ECIS Zθ, Applied BioPhysics, Troy, NY). When added to ECIS arrays and adhered to electrodes, cells behave as insulators, increasing impedance. In monolayers of confluent cells, measured impedance is primarily determined by morphology of the cells, whereas changes in impedance in stimulated cells are caused by changes of EB integrity (Tiruppathi et al., 1992; Szulcek et al., 2014). HUVECs and HULECs were seeded (2.0 × 10^5^ cells/well) and cultured at confluence in eight-well arrays (8WE10+, Applied BioPhysics, Troy, NY) coated with fibronectin (10 μg/mL) specific for this system. After confluence of cell monolayers to the ECIS arrays (normally after 48 h), cells were starved for 2 h, and stimulated with 20% serum from patients or healthy individuals. EB integrity was continuously monitored for 36 hcells.

### 2.7 Statistical analysis

Data are presented as mean ± standard deviation (SD) or as medians and interquartile range (IQR), as indicated. Differences in continuous variables were analyzed using *t*-test or Mann-Whitney test for comparison of two variables, or Anova when three or more variables were compared. Data distribution was assessed by the D’Agostino & Pearson normality test. Correlation was calculated using the Pearson or Spearman correlation coefficient. A *p*-value ≤0.05 was considered statistically significant. All statistical analysis were performed using SPSS version 26 (IBM) or GraphPad Prism 8.0 Software (GraphPad Inc.,).

## 3 Results

In total, 30 patients and 30 healthy individuals were included in the study. The characteristics of this cohort have been previously described (Henderson et al., 2022) and are summarized in Table 1.

**TABLE 1:** Clinical and laboratory characteristics of the study population.

	Patients (*n* = 30)	Healthy individuals (*n* = 30)	P
Age^a^	52.7 ± 12.3	50.3 ± 9.2	0.40
Sex, male:female	16:14	16:14	1.00
Body mass index^a^	30.6 ± 6.6	25.9 ± 4.2	0.006
Hemoglobin, g/dL^a^	13.96 ± 1.91	14.30 ± 1.11	0.42
Leukocytes, ^b^10^9^/L^a^	8.04 ± 3.91	5.58 ± 1.58	0.004
Neutrophils, ^b^10^9^/L^a^	6.38 ± 3.77	3.09 ± 0.93	<0.001
Lymphocytes, ^b^10^9^/L^a^	1.20 ± 0.55	1.79 ± 0.28	<0.001
Platelets, ^b^10^9^/L^a^	216.33 ± 93.02	245.59 ± 40.34	0.12
NLR^a^	6.19 ± 4.26	1.72 ± 0.61	<0.001
Time from symptom onset, days^b^	8.1 ± 2.3	NA	
CT score^b^	17.8 ± 7.3	NA	
Total hospital length of stay, days^b^	12.9 ± 9.8	NA	
Need for intensive care, yes (%)	12/30 (40%)	NA	
Length of intensive care stay, days	6.1 ± 9.7	NA	
Mortality	2/30	NA	

^a^ Mean ± SD; NLR, neutrophil:lymphocyte ratio.

^b^ After admission; NA, not applicable.

**FIGURE 1:**
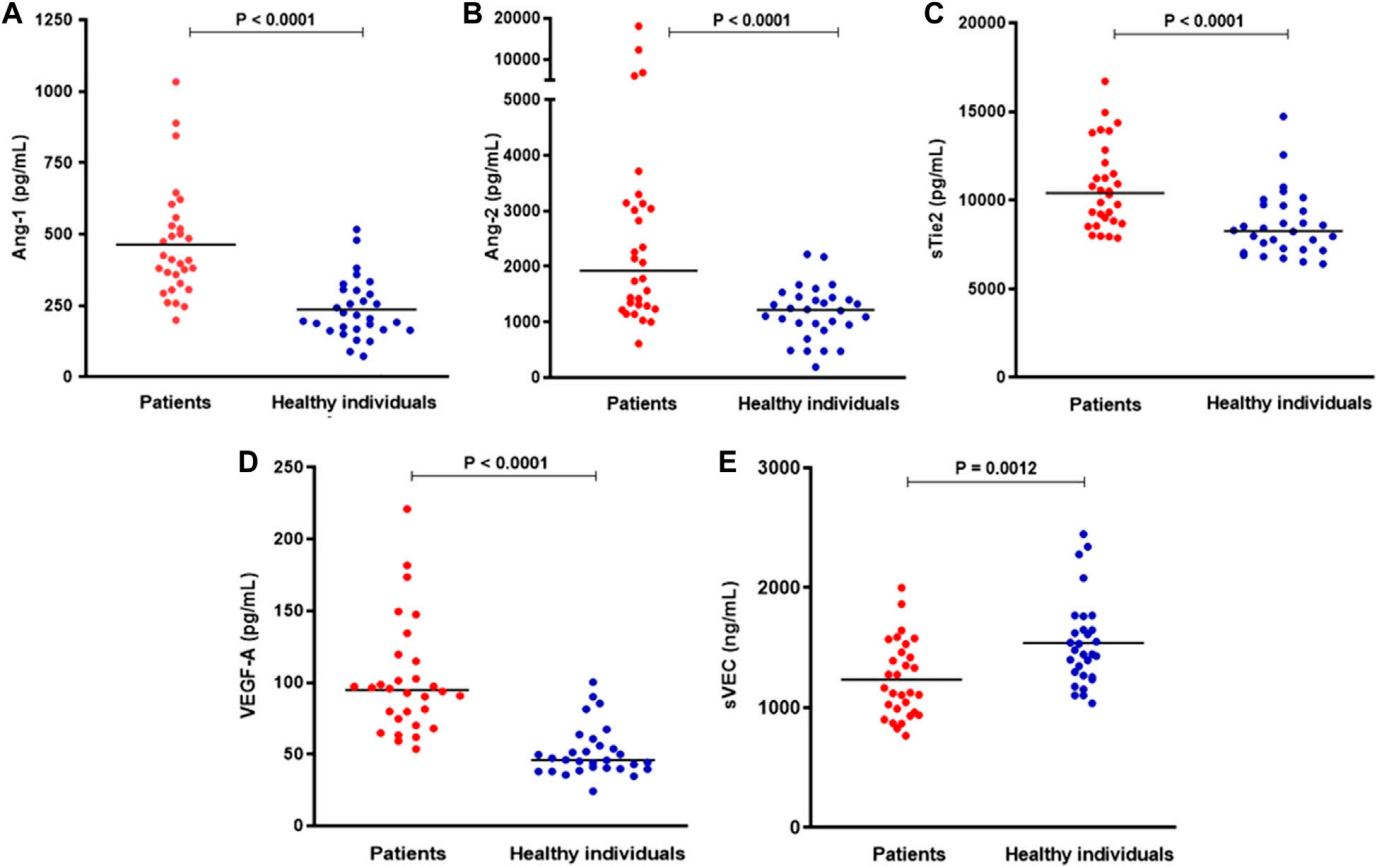
Plasma levels of **(A)** Ang-1, **(B)** Ang-2, **(C)** sTie2, **(D)** VEGF-A, and **(E)** sVEC in patients with COVID-19 and healthy individuals. Horizontal bars indicate median values, and *p* values are from Mann-Whitney test (*n* = 28–30 per group).

Patients with COVID-19 presented increased levels of Ang-1, Ang-2, sTie2 and VEGF-A when compared to healthy individuals (*p* > 0.0001). Patients with COVID-19 had decreased sVEC levels compared to healthy individuals (*p* = 0.0012) (Figure 1).

**FIGURE 2:**
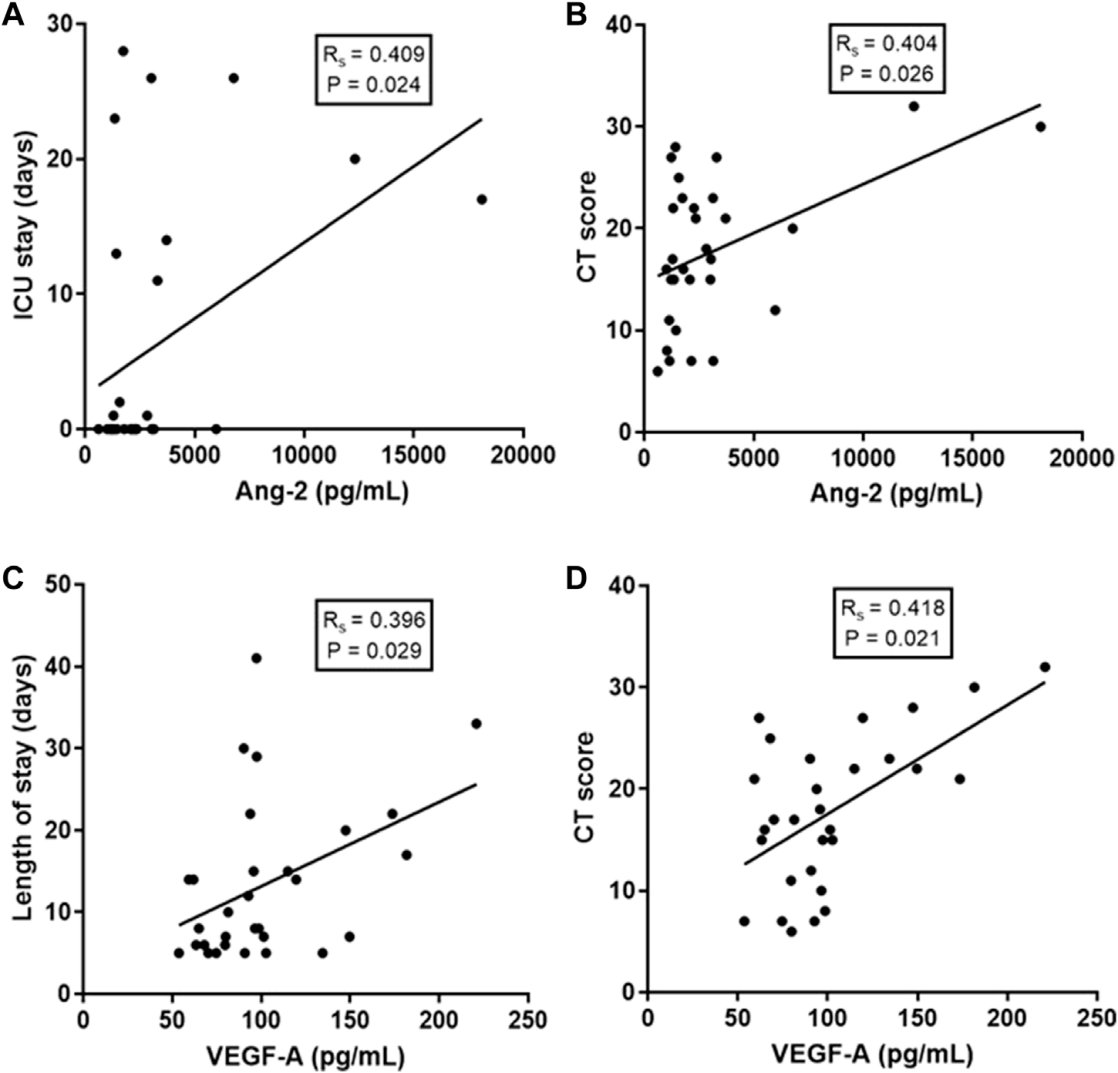
Correlations of EB integrity mediators with clinical and laboratory markers of COVID-19 severity. **(A)** Correlation of Ang-2 levels with ICU (intensive care unit) stay; **(B)** Correlation of Ang-2 levels with a lung CT (computerized tomography) score; **(C)** Correlation of VEGF-A levels with total hospital length of stay and **(D)** Correlation of VEGF-A levels with CT score. Spearman correlation coefficient.

Correlation analyses demonstrated that levels of some of these EB mediators were associated with clinical and laboratory markers of COVID-19 severity. Specifically, Ang-2 levels were associated with length of intensive care unit (ICU) stay (Rs = 0.409; *p* = 0.024) and with the extent of lung disease estimated by a CT score (Rs = 0.404; *p* = 0.026). Similarly, VEGF-A levels were associated with total hospital length of stay (LOS) (Rs = 0.396; *p* = 0.029) and with the extent of lung disease (Rs = 0.418; *p* = 0.021) (Figure 2). No additional significant correlation was observed between the other levels of EB mediators and other clinical parameters of disease severity.

**FIGURE 3:**
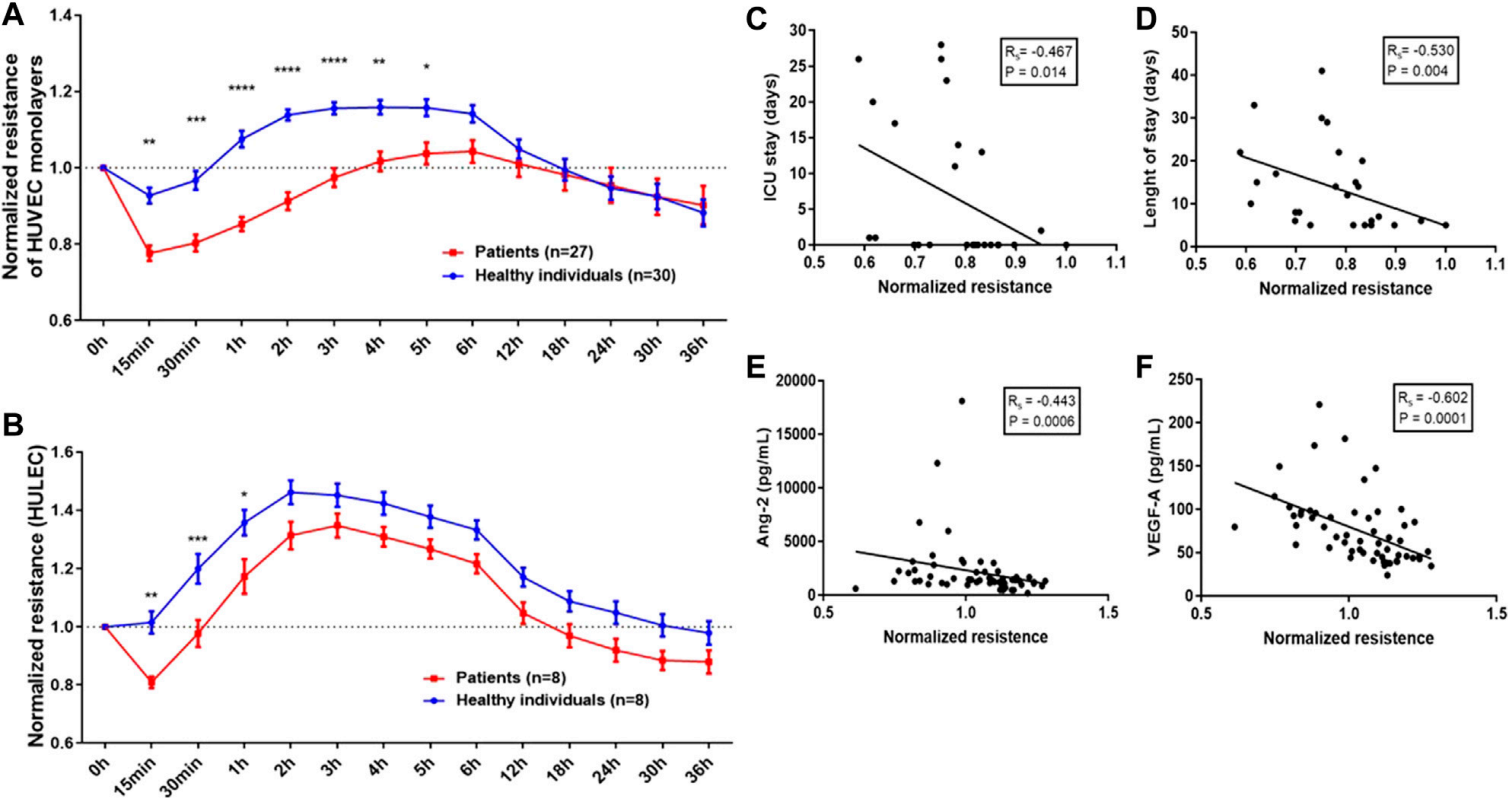
In **(A)** EB integrity of HUVEC monolayers upon stimulation by serum from COVID-19 patients and healthy individuals (*n* = 27–30 per group). The lower the normalized resistance, the higher the magnitude of EB disruption. Significant differences (* to****) are evident from 15 min to 5 h (Anova corrected for multiple comparisons). **(B)** EB integrity of HULEC monolayers upon stimulation by serum from COVID-19 patients and healthy individuals (*n* = 8 per group). Significant differences (* to***) are evident from 15 min to 1 h (Anova corrected for multiple comparisons). In **(C)** and **(D)**, we shown the correlation of EB disruption with clinically relevant outcomes such days of intensive care unit (ICU) stay and total hospital length of stay respectively. In **(E)** and **(F)**, Ang-2 and VEGF-A levels was correlated with EB disruption. (*n* = 57–60 per group). Negative correlations (Spearman test) indicate that the magnitude of EB disruption is associated with worse outcomes. *p* values: * <0.05; ** <0.01; *** <0.001; **** <0.0001.

We next evaluated whether serum from COVID-19 patients induced changes in EB integrity in monolayers of endothelial cells, using serum from healthy individuals as a control. As shown in Figure 3A, a significant decrease in EB integrity of HUVEC monolayers was evident as early as 15 min after serum stimulation, lasting up to 5 h. Similar data were obtained in HULECs (Figure 3B).

Finally, we explored whether the magnitude of EB disruption measured by ECIS was associated with clinically relevant outcomes, and whether it was dependent on circulating levels of mediators of the Ang/Tie2 or VEGF-A pathways. For these analyses we used data obtained 1 h after serum stimulation of endothelial cell monolayers. As shown in Figures 3C, D, significant correlations were observed between the magnitude of EB disruption with length of stay both in the ICU and in as total hospital LOS. Moreover, consistent correlations were observed between EB disruption and serum levels of Ang-2 and VEGF-A (Figures 3E, F).

## 4 Discussion

Both EB disruption and angiogenesis have been shown to contribute to the pathogenesis of inflammatory conditions such as sepsis (Park-windhol and Amore, 2016) and COVID-19 (Ackermann et al., 2020), being considered potential therapeutic targets in these conditions. In this context, the main contribution of our study was the demonstration that i) levels of soluble VEC were decreased, and of soluble Tie2 were increased in COVID-19, and ii) that EB disruption induced by serum from patients with COVID-19 is associated with clinical and laboratory outcomes, and correlated with levels of two key regulators of angiogenesis and EB integrity, Ang-2 and VEGF-A.

The fact that a specific cellular pathway can be both beneficial and detrimental in the pathogenesis of an inflammatory condition forms the basis of the immunothrombosis model (Engelmann and Massberg, 2013). Both EB disruption and angiogenesis are key components of the host response to an infection or tissue damage, as they allow the access of leukocytes to tissues and promote tissue repair (Seeley et al., 2012). Accordingly, circulating levels of mediators of EB integrity and angiogenesis such as VEGF-A and angiopoietins have been associated with the severity of sepsis and septic shock in humans (Alves et al., 2011; Flemming et al., 2015; Seol et al., 2020), and inhibition of these pathways have been shown to improve outcomes in animal models of sepsis and cellular models of COVID-19.

Here we first confirmed previous reports that Ang-2 and VEGF-A levels are increased in COVID-19 (Smadja et al., 2021; Vassiliou et al., 2021), extending these findings to include soluble Tie2 and soluble VEC levels. Significantly higher levels of sTie2 and significantly lower levels of sVEC were demonstrated. The former could be caused either by shedding of this receptor by cellular proteases or by an active secretion process, in a similar mechanism to that described for sFlt-1, in which this soluble form of the VEGF-A receptor functions as a decoy receptor, modulating the effects of its ligand (Kendall et al., 1996). Of note, higher sTie2 levels have been previously described in conditions such as acute myocardial infarction (Wang et al., 2005), acute myeloid leukemia (Attia et al., 2010), neuroendocrine tumors (Melen-Mucha et al., 2012) and obesity (Gozal et al., 2017), among other conditions associated with inflammation. Moreover, sVEC levels were lower in COVID-19, in an interesting contrast with sepsis (Zhang et al., 2010), autoimmune disease (Chen et al., 2014) and atherosclerosis (Soeki et al., 2004), in which this mediator was consistently higher in patients compared to healthy individuals. Of note, while to our knowledge no other study compared sVEC levels in COVID-19 with healthy individuals, a smaller study that compared levels of this mediator between COVID-19 survivors and non-survivors found lower VEC levels in the latter group (Vassiliou et al., 2021). Given the extensive disruption of the alvelo-capillary barrier in COVID-19 compared to sepsis and other inflammatory conditions, and the relevance of VEC internalization in EB regulation (Gavard, 2014), one could speculate that these contrasting results underlie differences in the pathogenesis of acute lung injury in COVID-19, which should be explored in future studies. A possible explanation could be the functional binding of sVEC to an inflamed vasculature and/or circulating cells, analogous to the mechanisms by which extracellular vesicles interact with cells (van Niel et al., 2018). Another notable difference between COVID-19 and sepsis is that the Ang-2/Ang-1 ratio did not increase in the former (data not shown), due to a concomitant and significant increase of the EB-stabilizing mediator Ang-1, observed in our patients. This pattern could represent a response to endothelial damage, and has been described by other authors in COVID-19 (Kaur et al., 2021; Hultström et al., 2022).

Our results also confirm previous reports about the association of Ang-2 and VEGF-A levels with clinical markers of disease severity in COVID-19, namely, length of ICU stay and extent of lung disease (Pine et al., 2020; Smadja et al., 2020; Hultström et al., 2022).

In addition, we explored the effect of sera from COVID-19 patients on EB integrity measured by ECIS, which is considered the gold standard for the *in vitro* exploration of EB integrity and has been used in key studies of EB biology (Moy et al., 2000; Umapathy et al., 2017; Robilliard et al., 2018). A significant decrease in EB integrity was observed as early as 15 min after serum stimulation, lasting until 5 h thereafter. The reversal of EB disruption after this time-points confirms the transient and functional (i.e., not associated with endothelial cell death) nature of this effect, which has also been observed in studies of sepsis and other inflammatory conditions using this model. The same pattern was confirmed in human lung microvascular endothelial cells, although the effect of serum was slightly less intense and more rapidly reversed. Although the biological relevance of these minor discrepancies cannot be confirmed, one possible explanation is the fact that EB in lungs have been reported to be relatively tighter than other organs (Claesson-Welsh et al., 2021). In fact, HUVECs have been shown to present a higher basal permeability than lung microvascular endothelial cells (van der Heijden et al., 2011). Finally, significant correlations were observed between the magnitude of EB disruption in this assay and clinical markers of disease severity, namely, length of hospital, and ICU stay. The effect of plasma from COVID-19 patients on EB integrity was explored for a recent study showed that effects of plasma from healthy subjects compared to moderate or severe COVID-19 cases resulted in increased endothelial permeability, causing loss of junctional VEC and formation of intercellular gaps within hours of exposure. Loss of junctional proteins has also been demonstrated in human lung tissue (Michalick et al., 2020).

Finally, we also report that the magnitude of this effect is associated with circulating levels of both Ang-2 and VEGF-A, which are well-known mediators of EB integrity during angiogenesis and inflammation both *in vitro* and *in vivo* (Folkman and Shing, 1992). Together our results reinforce the concept that EB is a relevant component of the pathogenesis of acute lung injury in COVID-19, and warrant additional mechanistic and interventional studies exploring the role of this process and their mediators in the pathogenesis and treatment of COVID-19 and other forms of viral acute lung injury.

Our study has limitations that need to be acknowledged. First, the sample size is relatively small, due to the limitation for recruitment in the clinical trial from which samples were obtained. On the other hand, the use of data from a clinical trial allowed us to obtain a consecutively recruited sample and high quality samples and clinical/outcome data. Moreover, sample size allowed us to demonstrate a series of relevant changes. Second, as an exploratory study, our results only demonstrate associations, which are not necessarily causal, and need to be explored in future studies, specifically designed for this purpose, addressing the cellular and molecular mechanisms of EB disruption in COVID-19.

## 5 Conclusion

In conclusion, levels of EB mediators are modulated in COVID-19, and associated with relevant clinical outcomes of disease severity. Moreover, we demonstrate for the first time that sTie and sVEC are also modulated (up- and down-regulated, respectively) in these patients. Finally, serum from these patients is capable to induce EB disruption in monolayers of endothelial cells, in a magnitude that is proportional to both disease severity and to circulating levels of Ang-2 and VEGF-A.

## Data Availability

The original contributions presented in the study are included in the article/Supplementary Material, further inquiries can be directed to the corresponding author.
